# Supplementary material for: Association of significant risk perception with the use of complementary and alternative medicine: A cross-sectional study in Hispanic patients with rheumatoid arthritis
Source: PLoS One. 2020 Aug 13;15(8):e0237504. doi: 10.1371/journal.pone.0237504 (PMC7425852; doi:10.1371/journal.pone.0237504)
Supplement: S1 Appendix — (PDF) [file pone.0237504.s001.pdf]

CUESTIONARIO DE PERCEPCIÓN DE RIESGO EN ARTRITIS REUMATOIDE

Fecha__________________

Estimado paciente, este cuestionario tiene la intención de conocer su opinión con respecto a algunas situaciones relacionadas con la artritis reumatoide, por lo que la información que contiene no es necesariamente la verdad sobre esta enfermedad.

Le pedimos que a cada pregunta o afirmación, nos ayude a **marcar sobre la línea de abajo** el punto que mejor refleje su opinión.

Ejemplo: 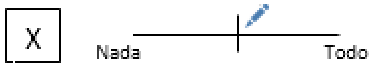

1 Seguramente siempre tendré dolor

Nada probable **Totalmente** probable

2 Las personas que tenemos artritis reumatoide siempre tendremos las articulaciones inflamadas.

Nada probable **Totalmente** probable

3 ¿Qué tan posible es que me sienta rígido y entumido por estar enfermo de artritis reumatoide?

Nada posible **Totalmente** posible

4 Las personas que estamos enfermos de artritis reumatoide siempre estaremos agotados

Nada probable **Totalmente** probable

5 ¿Qué tan posible es que me salgan bolitas (nódulos) en alguna parte del cuerpo por estar enfermo de artritis reumatoide?

Nada probable **Totalmente** probable

6 Seguramente en algún momento sentiré calambres, hormigueos y ardor en mis pies, piernas o brazos

Nada probable **Totalmente** probable

7 Puedo esperar que me salgan manchas porque mis venas se inflamaron y la sangre no circula adecuadamente

Nada probable **Totalmente** probable

8 A las personas con artritis reumatoide, se nos lastimará el cuello y nos dolerá la parte de atrás, por donde sale la joroba

Nada probable **Totalmente** probable

9 Puedo esperar que los ojos y la boca me molesten por sentirlos secos

Nada probable **Totalmente** probable

10 En algún momento voy a enfermarme de los ojos

Nada probable **Totalmente** probable

11 Enfermarme del corazón es algo que muy probablemente me pase

Nada probable **Totalmente** probable

12 Me voy a enfermar de los pulmones en algún momento

Nada probable **Totalmente** probable

13 Seguramente voy a tener problemas de la piel en algún momento

Nada probable **Totalmente** probable

14 La artritis reumatoide me llevará a la muerte

Nada probable **Totalmente** probable

15 Para tratar mi artritis necesitaré tomar muchos medicamentos durante largo tiempo y esto probablemente me cause problemas

Nada probable **Totalmente** probable

16 ¿Cuál es la probabilidad de que la artritis reumatoide me lleve a tener discapacidad?

Nada probable **Totalmente** probable

17 ¿Qué tan posible es que mis dedos se deformen debido a que tengo artritis reumatoide?

Nada posible **Totalmente** posible

18 ¿Qué tan probable es que mis articulaciones se lastimen y necesite que me pongan una articulación artificial? (prótesis)

Nada probable **Totalmente** probable

19 Sentirse triste, desanimado y sin futuro es algo que nos pasará a quienes tenemos artritis reumatoide

Nada probable **Totalmente** probable

20 La artritis reumatoide es una enfermedad que me llevará a depender de los demás y a perder mi autonomía

Nada probable **Totalmente** probable

21 ¿Qué tan posible es que pierda mi trabajo o pase por una crisis económica debido a que estoy enfermo de artritis reumatoide?

Nada posible **Totalmente** posible

22 ¿Qué tanto nos pasará a quienes tenemos artritis reumatoide, que las relaciones con nuestras parejas, familiares y/o amigos se verán afectadas de alguna manera?

Nada probable **Totalmente** probable

23 De cada 10 mujeres que se enferman de artritis reumatoide, ¿cuántas tendrán problemas para embarazarse o durante el embarazo y darán a luz hijos enfermos?

Ninguna **Todas**

24 ¿Qué tan responsable soy de las molestias y complicaciones que pueda tener por la artritis reumatoide?

Nada **Totalmente**

25 Creo que las molestias y complicaciones de la enfermedad que tengo, se pueden prevenir

Nada probable **Totalmente** probable

26 ¿Qué tan capaz me siento de controlar las molestias y complicaciones propias de mi enfermedad?

Nada **Totalmente**

27 ¿Qué tan grave considera usted una enfermedad como la artritis reumatoide?

Nada grave **Totalmente** grave

MUCHAS GRACIAS POR HABER CONTESTADO ESTE CUESTIONARIO

Risk perception questionnaire (RPQ)

# Item	Spanish version	English version
1	Seguramente siempre tendré dolor	I will always have pain
2	Las personas que tenemos artritis reumatoide siempre tendremos las articulaciones inflamadas.	People who have rheumatoid arthritis will always have swollen joints
3	¿Qué tan posible es que me sienta rígido y entumido por estar enfermo de artritis reumatoide?	How likely is it that I feel stiff and numb because I have rheumatoid arthritis?
4	Las personas que estamos enfermos de artritis reumatoide siempre estaremos agotados	People who are suffering from rheumatoid arthritis will always be exhausted
5	¿Qué tan posible es que me salgan bolitas (nódulos) en alguna parte del cuerpo por estar enfermo de artritis reumatoide?	How likely is it that I will get lumps (nodules) in some part of my body because I have rheumatoid arthritis?
6	Seguramente en algún momento sentiré calambres, hormigueos y ardor en mis pies, piernas o brazos	Surely at some point, I will feel cramps, tingling and burning in my feet, legs or arms
7	Puedo esperar que me salgan manchas porque mis venas se inflamaron y la sangre no circula adecuadamente	I can expect to get spots because my veins are swelling and blood is not circulating properly
8	A las personas con artritis reumatoide, se nos lastimará el cuello y nos dolerá la parte de atrás, por donde sale la joroba	People with rheumatoid arthritis will feel pain in the neck and the back of neck where the hump is
9	Puedo esperar que los ojos y la boca me molesten por sentirlos secos	I can expect my eyes and mouth to bother me because they feel dry
10	En algún momento voy a enfermarme de los ojos	At some point I'm going to experience eye disease
11	Enfermarme del corazón es algo que muy probablemente me pase	Heart disease is something that will probably happen to me
12	Me voy a enfermar de los pulmones en algún momento	I'm going to have lung disease at some point
13	Seguramente voy a tener problemas de la piel en algún momento	Surely I will have skin problems at some point
14	La artritis reumatoide me llevará a la muerte	Rheumatoid arthritis will kill me
15	Para tratar mi artritis necesitaré tomar muchos medicamentos durante largo tiempo y esto probablemente me cause problemas	To treat my arthritis I will need to take many medications for a long time and this will probably cause me problems
16	¿Cuál es la probabilidad de que la artritis reumatoide me lleve a tener discapacidad?	What is the probability that rheumatoid arthritis causes me disability?
17	¿Qué tan posible es que mis dedos se deformen debido a que tengo artritis reumatoide?	How possible is it that my fingers will become deformed because I have rheumatoid arthritis?
18	¿Qué tan probable es que mis articulaciones se lastimen y necesite que me pongan una articulación artificial? (prótesis)	How likely is it that my joints hurt and I will need to use an artificial joint? (prosthesis)
19	Sentirse triste, desanimado y sin futuro es algo que nos pasará a quienes tenemos artritis reumatoide	Feeling sad, discouraged and without a future is something that will happen to those of us with rheumatoid arthritis
20	La artritis reumatoide es una enfermedad que me llevará a depender de los demás y a perder mi autonomía	Rheumatoid arthritis is a disease that will make me depend on others and lose my autonomy

21	¿Qué tan posible es que pierda mi trabajo o pase por una crisis económica debido a que estoy enfermo de artritis reumatoide?	How possible is it that I will lose my job or have an economic crisis because I am sick with rheumatoid arthritis?
22	¿Qué tanto nos pasará a quienes tenemos artritis reumatoide, que las relaciones con nuestras parejas, familiares y/o amigos se verán afectadas de alguna manera?	How much will rheumatoid arthritis affect the relationships with our partners, family and / or friends in some way?
23	De cada 10 mujeres que se enferman de artritis reumatoide, ¿cuántas tendrán problemas para embarazarse o durante el embarazo y darán a luz hijos enfermos?	Of every 10 women who get rheumatoid arthritis, how many will have problems getting pregnant or during pregnancy and will give birth to sick children?
24	¿Qué tan responsable soy de las molestias y complicaciones que pueda tener por la artritis reumatoide?	How responsible I am for the complications I may have from rheumatoid arthritis?
25	Creo que las molestias y complicaciones de la enfermedad que tengo, se pueden prevenir	I think that the complications I may have due to my illness can be prevented
26	¿Qué tan capaz me siento de controlar las molestias y complicaciones propias de mi enfermedad?	How capable am I of controlling the discomfort and complications of my illness
27	¿Qué tan grave considera usted una enfermedad como la artritis reumatoide?	How serious do you consider a disease such as rheumatoid arthritis?

The VAS consisted of a 100-millimeter straight line with a verbal description at the endpoints. “No likelihood” appeared at the left side of the scale, and “Absolute likelihood” appeared at the right side of the scale.
