# Supplementary material for: Association of significant risk perception with the use of complementary and alternative medicine: A cross-sectional study in Hispanic patients with rheumatoid arthritis
Source: PLoS One. 2020 Aug 13;15(8):e0237504. doi: 10.1371/journal.pone.0237504 (PMC7425852; doi:10.1371/journal.pone.0237504)
Supplement: S2 Appendix — (PDF) [file pone.0237504.s002.pdf]

Cuestionario Internacional sobre Medicinas Alternativas y Complementarias (I-CAM-Q) – Versión Español

Introducción:

Le invitamos a responder un cuestionario.

A. Consultas a profesionales o expertos. Los problemas de salud pueden ser tratados por diferentes profesionales. Quisiera hacerle algunas preguntas sobre esto.

[PROFESIONAL o EXPERTO]	a. En los últimos 12 meses, consultó a un ________?	b. Cuántas veces consultó a un _____ en los últimos 3 meses?	c. Cuál fue el motivo *principal* por el que usted consultó a un ______ por *última vez*, fue por.....?	d. Cuán beneficioso le resultó ver a un _______? Diría que . . ?
1. Médico	□ 1. Si (Preguntar A1b)<br>□ 2. No (Pasar a A2)<br>□ 8. No sabe (Pasar a A2)<br>□ 9. No contesta (Pasar a A2)	□ 00. Ninguna<br>□ __ __ veces<br>□ 77. No corresponde<br>□ 88. No sabe<br>□ 99. No contesta	□ 1. Un problema de salud agudo, es decir, uno que duró menos de un mes?<br>□ 2. Un problema de salud crónico, es decir, uno que duró más de un mes, ya sea para tratar el problema en sí o sus síntomas?<br>□ 3. Para mejorar su bienestar<br>□ 4. Otra, por favor especificar: ________________________________<br>□ 7. No corresponde<br>□ 8. No sabe<br>□ 9. No contesta	□ 1. Muy beneficioso<br>□ 2. Algo beneficioso<br>□ 3. Para nada beneficioso<br>□ 7. No corresponde<br>□ 8. No sabe<br>□ 9. No contesta
2. Quiropráctico	□ 1. Si (Preguntar A2b)<br>□ 2. No (Pasar a A3)<br>□ 8. No sabe (Pasar a A3)<br>□ 9. No contesta (Pasar a A3)	□ 00. Ninguna<br>□ __ __ veces<br>□ 77. No corresponde<br>□ 88. No sabe<br>□ 99. No contesta	□ 1. Un problema de salud agudo, es decir, uno que duró menos de un mes?<br>□ 2. Un problema de salud crónico, es decir, uno que duró más de un mes, ya sea para tratar el problema en sí o sus síntomas?<br>□ 3. Para mejorar su bienestar<br>□ 4. Otra, por favor especificar: ________________________________<br>□ 7. No corresponde<br>□ 8. No sabe<br>□ 9. No contesta	□ 1. Muy beneficioso<br>□ 2. Algo beneficioso<br>□ 3. Para nada beneficioso<br>□ 7. No corresponde<br>□ 8. No sabe<br>□ 9. No contesta

3. Homeópata	□ 1. Si (Preguntar A3b)<br>□ 2. No (Pasar a A4)<br>□ 8. No sabe (Pasar a A4)<br>□ 9. No contesta (Pasar a A4)	□ 00. Ninguna<br>□ __ __ veces<br>□ 77. No corresponde<br>□ 88. No sabe<br>□ 99. No contesta	□ 1. Un problema de salud agudo, es decir, uno que duró menos de un mes?<br>□ 2. Un problema de salud crónico, es decir, uno que duró más de un mes, ya sea para tratar el problema en sí o sus síntomas?<br>□ 3. Para mejorar su bienestar<br>□ 4. Otra, por favor especificar: ________________________________<br>□ 7. No corresponde<br>□ 8. No sabe<br>□ 9. No contesta	□ 1. Muy beneficioso<br>□ 2. Algo beneficioso<br>□ 3. Para nada beneficioso<br>□ 7. No corresponde<br>□ 8. No sabe<br>□ 9. No contesta
[PROFESIONAL O EXPERTO]	a. En los últimos 12 meses, consultó a un ________?	b. Cuántas veces consultó a un _____ en los últimos 3 meses?	c. Cuál fue el motivo *principal* por el que usted consultó a un ______ por *última vez*, fue por.....?	d. Cuán beneficioso le resultó ver a un _______? Diría que . . ?
4. Acupunturista	□ 1. Si (Preguntar A4b)<br>□ 2. No (Pasar a A5)<br>□ 8. No sabe (Pasar a A5)<br>□ 9. No contesta (Pasar a A5)	□ 00. Ninguna<br>□ __ __ veces<br>□ 77. No corresponde<br>□ 88. No sabe<br>□ 99. No contesta	□ 1. Un problema de salud agudo, es decir, uno que duró menos de un mes?<br>□ 2. Un problema de salud crónico, es decir, uno que duró más de un mes, ya sea para tratar el problema en sí o sus síntomas?<br>□ 3. Para mejorar su bienestar<br>□ 4. Otra, por favor especificar: ________________________________<br>□ 7. No corresponde<br>□ 8. No sabe<br>□ 9. No contesta	□ 1. Muy beneficioso<br>□ 2. Algo beneficioso<br>□ 3. Para nada beneficioso<br>□ 7. No corresponde<br>□ 8. No sabe<br>□ 9. No contesta
5. Fitoterapeuta o Herbalista (Un experto o profesional que receta yuyos, infusiones, tinturas y/o plantas medicinales incluyendo tratamientos con flores)	□ 1. Si (Preguntar A5b)<br>□ 2. No (Pasar a A6)<br>□ 8. No sabe (Pasar a A6)<br>□ 9. No contesta (Pasar a A6)	□ 00. Ninguna<br>□ __ __ veces<br>□ 77. No corresponde<br>□ 88. No sabe<br>□ 99. No contesta	□ 1. Un problema de salud agudo, es decir, uno que duró menos de un mes?<br>□ 2. Un problema de salud crónico, es decir, uno que duró más de un mes, ya sea para tratar el problema en sí o sus síntomas?<br>□ 3. Para mejorar su bienestar<br>□ 4. Otra, por favor especificar: ________________________________<br>□ 7. No corresponde<br>□ 8. No sabe<br>□ 9. No contesta	□ 1. Muy beneficioso<br>□ 2. Algo beneficioso<br>□ 3. Para nada beneficioso<br>□ 7. No corresponde<br>□ 8. No sabe<br>□ 9. No contesta
6. Sanador espiritual	□ 1. Si (Preguntar A6b)<br>□ 2. No (Pasar a A7)<br>□ 8. No sabe (Pasar a A7)<br>□ 9. No contesta (Pasar a A7)	□ 00. Ninguna<br>□ __ __ veces<br>□ 77. No corresponde<br>□ 88. No sabe<br>□ 99. No contesta	□ 1. Un problema de salud agudo, es decir, uno que duró menos de un mes?<br>□ 2. Un problema de salud crónico, es decir, uno que duró más de un mes, ya sea para tratar el problema en sí o sus síntomas?<br>□ 3. Para mejorar su bienestar	□ 1. Muy beneficioso<br>□ 2. Algo beneficioso<br>□ 3. Para nada beneficioso<br>□ 7. No corresponde<br>□ 8. No sabe<br>□ 9. No contesta

			□ 4. Otra, por favor especificar: ________________________________<br>□ 7. No corresponde<br>□ 8. No sabe<br>□ 9. No contesta	
[PROFESIONAL o EXPERTO]	a. En los últimos 12 meses, consultó a un ________?	b. Cuántas veces consultó a un _____ en los últimos 3 meses?	c. Cuál fue el motivo *principal* por el que usted consultó a un ______ por *última vez*, fue por.....?	d. Cuán beneficioso le resultó ver a un _______? Diría que . . ?
7. Ha visitado a algún otro profesional o experto en los últimos 12 meses?<br><br>Qué tipo de profesional ha visitado?<br>_______________	□ 1. Si (Preguntar A7b)<br>□ 2. No (Pasar a Sección B)<br>□ 8. No sabe (Pasar a Sección B)<br>□ 9. No contesta (Pasar a Sección B)	□ 00. Ninguna<br>□ __ __ veces<br>□ 77. No corresponde<br>□ 88. No sabe<br>□ 99. No contesta	□ 1. Un problema de salud agudo, es decir, uno que duró menos de un mes?<br>□ 2. Un problema de salud crónico, es decir, uno que duró más de un mes, ya sea para tratar el problema en sí o sus síntomas?<br>□ 3. Para mejorar su bienestar<br>□ 4. Otra, por favor especificar: ________________________________<br>□ 7. No corresponde<br>□ 8. No sabe<br>□ 9. No contesta	□ 1. Muy beneficioso<br>□ 2. Algo beneficioso<br>□ 3. Para nada beneficioso<br>□ 7. No corresponde<br>□ 8. No sabe<br>□ 9. No contesta

B. *Tratamientos realizados por Médicos.* Quisiera preguntarle sobre tratamientos que haya recibido de parte de un médico en los últimos 12 meses. [SI EL ENTREVISTADO NO HA VISITADO A UN MÉDICO EN LOS ÚLTIMOS 12 MESES, PASAR A LA SECCIÓN C]

[TRATAMIENTO]	a. En los últimos 12 meses, ha recibido ______ de parte de un médico?	b. Cuántas veces recibió _____ de parte de un médico en los últimos 3 meses?	c. Cuál fue el motivo *principal* por el que recibió ______ de parte de un médico por *última vez*? Fue ……?	d. Cuán beneficioso le resultó haber recibido _______ de parte de un médico? Diría que…..?
1. Manipulación articular y/o masajes	□ 1. Si (Preguntar B1b)<br>□ 2. No (Pasar a B2)<br>□ 8. No sabe (Pasar a B2)<br>□ 9. No contesta (Pasar a B2)	□ 00. Ninguna<br>__ __ veces<br>□ 77. No corresponde<br>□ 88. No sabe<br>□ 99. No contesta	□ 1. Un problema de salud agudo, es decir, uno que duró menos de un mes?<br>□ 2. Un problema de salud crónico, es decir, uno que duró más de un mes, ya sea para tratar el problema en sí o sus síntomas?<br>□ 3. Para mejorar su bienestar<br>□ 4. Otra, por favor especificar: ______________________________<br>□ 7. No corresponde<br>□ 8. No sabe<br>□ 9. No contesta	□ 1. Muy beneficioso<br>□ 2. Algo beneficioso<br>□ 3. Para nada beneficioso<br>□ 7. No corresponde<br>□ 8. No sabe<br>□ 9. No contesta
2. Homeopatía	□ 1. Si (Preguntar B2b)<br>□ 2. No (Pasar a B3)<br>□ 8. No sabe (Pasar a B3)<br>□ 9. No contesta (Pasar a B3)	□ 00. Ninguna<br>__ __ veces<br>□ 77. No corresponde<br>□ 88. No sabe<br>□ 99. No contesta	□ 1. Un problema de salud agudo, es decir, uno que duró menos de un mes?<br>□ 2. Un problema de salud crónico, es decir, uno que duró más de un mes, ya sea para tratar el problema en sí o sus síntomas?<br>□ 3. Para mejorar su bienestar<br>□ 4. Otra, por favor especificar: ______________________________<br>□ 7. No corresponde<br>□ 8. No sabe<br>□ 9. No contesta	□ 1. Muy beneficioso<br>□ 2. Algo beneficioso<br>□ 3. Para nada beneficioso<br>□ 7. No corresponde<br>□ 8. No sabe<br>□ 9. No contesta
3. Acupuntura	□ 1. Si (Preguntar B3b)<br>□ 2. No (Pasar a B4)<br>□ 8. No sabe (Pasar a B4)<br>□ 9. No contesta (Pasar a B4)	□ 00. Ninguna<br>__ __ veces<br>□ 77. No corresponde<br>□ 88. No sabe<br>□ 99. No contesta	□ 1. Un problema de salud agudo, es decir, uno que duró menos de un mes?<br>□ 2. Un problema de salud crónico, es decir, uno que duró más de un mes, ya sea para tratar el problema en sí o sus síntomas?<br>□ 3. Para mejorar su bienestar<br>□ 4. Otra, por favor especificar: ______________________________<br>□ 7. No corresponde<br>□ 8. No sabe<br>□ 9. No contesta	□ 1. Muy beneficioso<br>□ 2. Algo beneficioso<br>□ 3. Para nada beneficioso<br>□ 7. No corresponde<br>□ 8. No sabe<br>□ 9. No contesta

[TRATAMIENTO]	a. En los últimos 12 meses, ha recibido ______ de parte de un médico?	b. Cuántas veces recibió _____ de parte de un médico en los últimos 3 meses?	c. Cuál fue el motivo *principal* por el que recibió ______ de parte de un médico por *última vez*? Fue ……?	d. Cuán beneficioso le resultó haber recibido _______ de parte de un médico? Diría que…..?
4. Hierbas, Yuyos, Infusiones o Tinturas	□ 1. Si (Preguntar B4b)<br>□ 2. No (Pasar a B5)<br>□ 8. No sabe (Pasar a B5)<br>□ 9. No contesta (Pasar a B5)	□ 00. Ninguna<br>__ __ veces<br>□ 77. No corresponde<br>□ 88. No sabe<br>□ 99. No contesta	□ 1. Un problema de salud agudo, es decir, uno que duró menos de un mes?<br>□ 2. Un problema de salud crónico, es decir, uno que duró más de un mes, ya sea para tratar el problema en sí o sus síntomas?<br>□ 3. Para mejorar su bienestar<br>□ 4. Otra, por favor especificar:<br>________________________________<br>□ 7. No corresponde<br>□ 8. No sabe<br>□ 9. No contesta	□ 1. Muy beneficioso<br>□ 2. Algo beneficioso<br>□ 3. Para nada beneficioso<br>□ 7. No corresponde<br>□ 8. No sabe<br>□ 9. No contesta
5. Sanación espiritual	□ 1. Si (Preguntar B5b)<br>□ 2. No (Pasar a B6)<br>□ 8. No sabe (Pasar a B6)<br>□ 9. No contesta (Pasar a B6)	□ 00. Ninguna<br>__ __ veces<br>□ 77. No corresponde<br>□ 88. No sabe<br>□ 99. No contesta	□ 1. Un problema de salud agudo, es decir, uno que duró menos de un mes?<br>□ 2. Un problema de salud crónico, es decir, uno que duró más de un mes, ya sea para tratar el problema en sí o sus síntomas?<br>□ 3. Para mejorar su bienestar<br>□ 4. Otra, por favor especificar:<br>________________________________<br>□ 7. No corresponde<br>□ 8. No sabe<br>□ 9. No contesta	□ 1. Muy beneficioso<br>□ 2. Algo beneficioso<br>□ 3. Para nada beneficioso<br>□ 7. No corresponde<br>□ 8. No sabe<br>□ 9. No contesta
6. Ha recibido algún otro tipo de tratamiento de parte de un médico en los últimos 12 meses?<br><br>Qué tipo de tratamiento ha recibido?<br>_______________	□ 1. Si (Preguntar B6b)<br>□ 2. No (Pasar a Sección C)<br>□ 8. No sabe (Pasar a Sección C)<br>□ 9. No contesta (Pasar a Sección C)	□ 00. Ninguna<br>__ __ veces<br>□ 77. No corresponde<br>□ 88. No sabe<br>□ 99. No contesta	□ 1. Un problema de salud agudo, es decir, uno que duró menos de un mes?<br>□ 2. Un problema de salud crónico, es decir, uno que duró más de un mes, ya sea para tratar el problema en sí o sus síntomas?<br>□ 3. Para mejorar su bienestar<br>□ 4. Otra, por favor especificar:<br>________________________________<br>□ 7. No corresponde<br>□ 8. No sabe<br>□ 9. No contesta	□ 1. Muy beneficioso<br>□ 2. Algo beneficioso<br>□ 3. Para nada beneficioso<br>□ 7. No corresponde<br>□ 8. No sabe<br>□ 9. No contesta

C. Uso de medicamentos a base de hierbas y suplementos dietarios. Además de los medicamentos que prescriben los médicos, algunas personas utilizan una gran variedad de otros productos para su salud, como por ejemplo, medicamentos a base de hierbas (yuyos) y suplementos dietéticos, que pueden ser comprimidos, cápsulas o líquidos (tés, tinturas, jarabes, etc.)

C1. HIERBAS (YUYOS)	a. En los últimos 12 meses que hierbas ha utilizado? [Si ninguna, pasar a C2]	b. Utiliza actualmente ___________?	c. Cuál fue la principal razón por la que utilizó______ por última vez? Fue por . . .?	d. Cuán beneficioso le resultó _______?<br>Diría que . . . ?
Hierba 1.<br>____________	□ 1. Si (Preguntar C1.1b)<br>□ 2. No (Pasar a C1.2)<br>□ 8. No sabe (Pasar a C1.2)<br>□ 9. No contesta (Pasar a C1.2)	□ 00. Ninguna<br>__ __ veces<br>□ 77. No corresponde<br>□ 88. No sabe<br>□ 99. No contesta	□ 1. Un problema de salud agudo, es decir, uno que duró menos de un mes?<br>□ 2. Un problema de salud crónico, es decir, uno que duró más de un mes, ya sea para tratar el problema en sí o sus síntomas?<br>□ 3. Para mejorar su bienestar<br>□ 4. Otra, por favor especificar:<br>________________________________<br>□ 7. No corresponde<br>□ 8. No sabe<br>□ 9. No contesta	□ 1. Muy beneficioso<br>□ 2. Algo beneficioso<br>□ 3. Para nada beneficioso<br>□ 7. No corresponde<br>□ 8. No sabe<br>□ 9. No contesta
Hierba 2.<br>____________	□ 1. Si (Preguntar C1.2b)<br>□ 2. No (Pasar a C2)<br>□ 8. No sabe (Pasar a C2)<br>□ 9. No contesta (Pasar a C2)	□ 00. Ninguna<br>__ __ veces<br>□ 77. No corresponde<br>□ 88. No sabe<br>□ 99. No contesta	□ 1. Un problema de salud agudo, es decir, uno que duró menos de un mes?<br>□ 2. Un problema de salud crónico, es decir, uno que duró más de un mes, ya sea para tratar el problema en sí o sus síntomas?<br>□ 3. Para mejorar su bienestar<br>□ 4. Otra, por favor especificar:<br>________________________________<br>□ 7. No corresponde<br>□ 8. No sabe<br>□ 9. No contesta	□ 1. Muy beneficioso<br>□ 2. Algo beneficioso<br>□ 3. Para nada beneficioso<br>□ 7. No corresponde<br>□ 8. No sabe<br>□ 9. No contesta

C2. VITAMINAS / MINERALES	a. En los últimos 12 meses, qué vitaminas o minerales ha utilizado?<br>[Si ninguna, pasar a C3]	b. Utiliza actualmente ___________?	c. Cuál fue la principal razón por la que utilizó______ por última vez? Fue por . . .?	d. Cuán beneficioso le resultó _______?<br>Diría que . . . ?
Vitamina/mineral 1.<br>____________	□ 1. Si (Preguntar C2.1b)<br>□ 2. No (Pasar a C2.2)<br>□ 8. No sabe (Pasar a C2.2)<br>□ 9. No contesta (Pasar a C2.2)	□ 00. Ninguna<br>__ __ veces<br>□ 77. No corresponde<br>□ 88. No sabe<br>□ 99. No contesta	□ 1. Un problema de salud agudo, es decir, uno que duró menos de un mes?<br>□ 2. Un problema de salud crónico, es decir, uno que duró más de un mes, ya sea para tratar el problema en sí o sus síntomas?<br>□ 3. Para mejorar su bienestar<br>□ 4. Otra, por favor especificar:<br>________________________________<br>□ 7. No corresponde<br>□ 8. No sabe<br>□ 9. No contesta	□ 1. Muy beneficioso<br>□ 2. Algo beneficioso<br>□ 3. Para nada beneficioso<br>□ 7. No corresponde<br>□ 8. No sabe<br>□ 9. No contesta
Vitamina/mineral 2.<br>____________	□ 1. Si (Preguntar C2.2b)<br>□ 2. No (Pasar a C3)<br>□ 8. No sabe (Pasar a C3)<br>□ 9. No contesta (Pasar a C3)	□ 00. Ninguna<br>__ __ veces<br>□ 77. No corresponde<br>□ 88. No sabe<br>□ 99. No contesta	□ 1. Un problema de salud agudo, es decir, uno que duró menos de un mes?<br>□ 2. Un problema de salud crónico, es decir, uno que duró más de un mes, ya sea para tratar el problema en sí o sus síntomas?<br>□ 3. Para mejorar su bienestar<br>□ 4. Otra, por favor especificar:<br>________________________________<br>□ 7. No corresponde<br>□ 8. No sabe<br>□ 9. No contesta	□ 1. Muy beneficioso<br>□ 2. Algo beneficioso<br>□ 3. Para nada beneficioso<br>□ 7. No corresponde<br>□ 8. No sabe<br>□ 9. No contesta

C3. REMEDIOS HOMEOPÁTICOS	a. En los últimos 12 meses, que remedios homeopáticos a utilizado?<br>[Si ninguno, finalice la entrevista]	b. Utiliza actualmente ___________?	c. Cuál fue la principal razón por la que utilizó______ por última vez? Fue por . . .?	d. Cuán beneficioso le resultó _______?<br>Diría que . . . ?
Remedio homeopático<br>1. ____________	□ 1. Si (Preguntar C3.1b)<br>□ 2. No (Pasar a sección D)<br>□ 8. NO SABE (PASAR A C3.2)<br>□ 9. NO CONTESTA (PASAR A C3.2)	□ 00. Ninguna<br>__ __ veces<br>□ 77. No corresponde<br>□ 88. No sabe<br>□ 99. No contesta	□ 1. Un problema de salud agudo, es decir, uno que duró menos de un mes?<br>□ 2. Un problema de salud crónico, es decir, uno que duró más de un mes, ya sea para tratar el problema en sí o sus síntomas?<br>□ 3. Para mejorar su bienestar<br>□ 4. Otra, por favor especificar:<br>________________________________<br>□ 7. No corresponde<br>□ 8. No sabe<br>□ 9. No contesta	□ 1. Muy beneficioso<br>□ 2. Algo beneficioso<br>□ 3. Para nada beneficioso<br>□ 7. No corresponde<br>□ 8. No sabe<br>□ 9. No contesta
Remedio homeopático<br>2. ____________	□ 1. Si (PREGUNTAR D3.2b)<br>□ 2. No (Pasar a sección D)<br>□ 8. NO SABE (Pasar a sección D)<br>□ 9. NO CONTESTA (Pasar a sección D)	□ 00. Ninguna<br>__ __ veces<br>□ 77. No corresponde<br>□ 88. No sabe<br>□ 99. No contesta	□ 1. Un problema de salud agudo, es decir, uno que duró menos de un mes?<br>□ 2. Un problema de salud crónico, es decir, uno que duró más de un mes, ya sea para tratar el problema en sí o sus síntomas?<br>□ 3. Para mejorar su bienestar<br>□ 4. Otra, por favor especificar:<br>________________________________<br>□ 7. No corresponde<br>□ 8. No sabe<br>□ 9. No contesta	□ 1. Muy beneficioso<br>□ 2. Algo beneficioso<br>□ 3. Para nada beneficioso<br>□ 7. No corresponde<br>□ 8. No sabe<br>□ 9. No contesta

D. Prácticas personales que promueven el bienestar. Además de los medicamentos que prescriben los médicos, algunas personas suelen utilizar diferentes tipos de prácticas personales para mejorar su salud.

[PRÁCTICAS PERSONALES]	a. En los últimos 12 meses, ha utilizado ________ como práctica personal?	b. Cuántas veces en los últimos 3 meses ha utilizado _______ como práctica personal?	c. Cuál fue la razón *principal* por la que utilizó _________ ? Fue….?	d. Cuán beneficioso le resultó _______?<br>Diría que . . . ?
1. Meditación	□ 1. Si (Preguntar D1b)<br>□ 2. No (Pasar a D2)<br>□ 8. No sabe (Pasar a D2)<br>□ 9. No contesta (Pasar a D2)	□ 00. Ninguna<br>__ __ veces<br>□ 77. No corresponde<br>□ 88. No sabe<br>□ 99. No contesta	□ 1. Un problema de salud agudo, es decir, uno que duró menos de un mes?<br>□ 2. Un problema de salud crónico, es decir, uno que duró más de un mes, ya sea para tratar el problema en sí o sus síntomas?<br>□ 3. Para mejorar su bienestar<br>□ 4. Otra, por favor especificar: ___________________________<br>□ 7. No corresponde<br>□ 8. No sabe<br>□ 9. No contesta	□ 1. Muy beneficioso<br>□ 2. Algo beneficioso<br>□ 3. Para nada beneficioso<br>□ 7. No corresponde<br>□ 8. No sabe<br>□ 9. No contesta
2. Yoga	□ 1. Si (Preguntar D2b)<br>□ 2. No (Pasar a D3)<br>□ 8. No sabe (Pasar a D3)<br>□ 9. No contesta (Pasar a D3)	□ 00. Ninguna<br>__ __ veces<br>□ 77. No corresponde<br>□ 88. No sabe<br>□ 99. No contesta	□ 1. Un problema de salud agudo, es decir, uno que duró menos de un mes?<br>□ 2. Un problema de salud crónico, es decir, uno que duró más de un mes, ya sea para tratar el problema en sí o sus síntomas?<br>□ 3. Para mejorar su bienestar<br>□ 4. Otra, por favor especificar: ____________________________<br>□ 7. No corresponde<br>□ 8. No sabe<br>□ 9. No contesta	□ 1. Muy beneficioso<br>□ 2. Algo beneficioso<br>□ 3. Para nada beneficioso<br>□ 7. No corresponde<br>□ 8. No sabe<br>□ 9. No contesta
3. Qi Gong (Chi Kung)	□ 1. Si (Preguntar D3b)<br>□ 2. No (Pasar a D4)<br>□ 8. No sabe (Pasar a D4)<br>□ 9. No contesta (Pasar a D4)	□ 00. Ninguna<br>__ __ veces<br>□ 77. No corresponde<br>□ 88. No sabe<br>□ 99. No contesta	□ 1. Un problema de salud agudo, es decir, uno que duró menos de un mes?<br>□ 2. Un problema de salud crónico, es decir, uno que duró más de un mes, ya sea para tratar el problema en sí o sus síntomas?<br>□ 3. Para mejorar su bienestar<br>□ 4. Otra, por favor especificar: ____________________________<br>□ 7. No corresponde<br>□ 8. No sabe<br>□ 9. No contesta	□ 1. Muy beneficioso<br>□ 2. Algo beneficioso<br>□ 3. Para nada beneficioso<br>□ 7. No corresponde<br>□ 8. No sabe<br>□ 9. No contesta

[PRÁCTICAS PERSONALES]	a. En los últimos 12 meses, ha utilizado ________ como práctica personal?	b. Cuántas veces en los últimos 3 meses ha utilizado _______ como práctica personal?	c. Cuál fue la razón *principal* por la que utilizó _________ ? Fue….?	d. Cuán beneficioso le resultó _______?<br>Diría que . . . ?
4. Tai Ji Quan (Tai Chi Chuan)	□ 1. Si (Preguntar D4b)<br>□ 2. No (Pasar a D5)<br>□ 8. No sabe (Pasar a D5)<br>□ 9. No contesta (Pasar a D5)	□ 00. Ninguna<br>__ __ veces<br>□ 77. No corresponde<br>□ 88. No sabe<br>□ 99. No contesta	□ 1. Un problema de salud agudo, es decir, uno que duró menos de un mes?<br>□ 2. Un problema de salud crónico, es decir, uno que duró más de un mes, ya sea para tratar el problema en sí o sus síntomas?<br>□ 3. Para mejorar su bienestar<br>□ 4. Otra, por favor especificar: ________________________________<br>□ 7. No corresponde<br>□ 8. No sabe<br>□ 9. No contesta	□ 1. Muy beneficioso<br>□ 2. Algo beneficioso<br>□ 3. Para nada beneficioso<br>□ 7. No corresponde<br>□ 8. No sabe<br>□ 9. No contesta
5. Técnicas de relajación	□ 1. Si (Preguntar D5b)<br>□ 2. No (Pasar a D6)<br>□ 8. No sabe (Pasar a D6)<br>□ 9. No contesta (Pasar a D6)	□ 00. Ninguna<br>__ __ veces<br>□ 77. No corresponde<br>□ 88. No sabe<br>□ 99. No contesta	□ 1. Un problema de salud agudo, es decir, uno que duró menos de un mes?<br>□ 2. Un problema de salud crónico, es decir, uno que duró más de un mes, ya sea para tratar el problema en sí o sus síntomas?<br>□ 3. Para mejorar su bienestar<br>□ 4. Otra, por favor especificar: ________________________________<br>□ 7. No corresponde<br>□ 8. No sabe<br>□ 9. No contesta	□ 1. Muy beneficioso<br>□ 2. Algo beneficioso<br>□ 3. Para nada beneficioso<br>□ 7. No corresponde<br>□ 8. No sabe<br>□ 9. No contesta
6. Visualización	□ 1. Si (Preguntar D6b)<br>□ 2. No (Pasar a D7)<br>□ 8. No sabe (Pasar a D7)<br>□ 9. No contesta (Pasar a D7)	□ 00. Ninguna<br>__ __ veces<br>□ 77. No corresponde<br>□ 88. No sabe<br>□ 99. No contesta	□ 1. Un problema de salud agudo, es decir, uno que duró menos de un mes?<br>□ 2. Un problema de salud crónico, es decir, uno que duró más de un mes, ya sea para tratar el problema en sí o sus síntomas?<br>□ 3. Para mejorar su bienestar<br>□ 4. Otra, por favor especificar: ________________________________<br>□ 7. No corresponde<br>□ 8. No sabe<br>□ 9. No contesta	□ 1. Muy beneficioso<br>□ 2. Algo beneficioso<br>□ 3. Para nada beneficioso<br>□ 7. No corresponde<br>□ 8. No sabe<br>□ 9. No contesta

[PRÁCTICAS PERSONALES]	a. En los últimos 12 meses, ha utilizado ________ como práctica personal?	b. Cuántas veces en los últimos 3 meses ha utilizado _______ como práctica personal?	c. Cuál fue la razón *principal* por la que utilizó _________ ? Fue….?	d. Cuán beneficioso le resultó _______?<br>Diría que . . . ?
7. Participar de una ceremonia de sanación tradicional	□ 1. Si (Preguntar D7b)<br>□ 2. No (Pasar a D8)<br>□ 8. No sabe (Pasar a D8)<br>□ 9. No contesta (Pasar a D8)	□ 00. Ninguna<br>__ __ veces<br>□ 77. No corresponde<br>□ 88. No sabe<br>□ 99. No contesta	□ 1. Un problema de salud agudo, es decir, uno que duró menos de un mes?<br>□ 2. Un problema de salud crónico, es decir, uno que duró más de un mes, ya sea para tratar el problema en sí o sus síntomas?<br>□ 3. Para mejorar su bienestar<br>□ 4. Otra, por favor especificar: ________________________________<br>□ 7. No corresponde<br>□ 8. No sabe<br>□ 9. No contesta	□ 1. Muy beneficioso<br>□ 2. Algo beneficioso<br>□ 3. Para nada beneficioso<br>□ 7. No corresponde<br>□ 8. No sabe<br>□ 9. No contesta
8. Rezar por su salud	□ 1. Si (Preguntar D8b)<br>□ 2. No (Pasar a D9)<br>□ 8. No sabe (Pasar a D9)<br>□ 9. No contesta (Pasar a D9)	□ 00. Ninguna<br>__ __ veces<br>□ 77. No corresponde<br>□ 88. No sabe<br>□ 99. No contesta	□ 1. Un problema de salud agudo, es decir, uno que duró menos de un mes?<br>□ 2. Un problema de salud crónico, es decir, uno que duró más de un mes, ya sea para tratar el problema en sí o sus síntomas?<br>□ 3. Para mejorar su bienestar<br>□ 4. Otra, por favor especificar: ________________________________<br>□ 7. No corresponde<br>□ 8. No sabe<br>□ 9. No contesta	□ 1. Muy beneficioso<br>□ 2. Algo beneficioso<br>□ 3. Para nada beneficioso<br>□ 7. No corresponde<br>□ 8. No sabe<br>□ 9. No contesta
10. Utilizó alguna otra práctica personal (sin contar medicamentos a base de hierbas o suplementos dietéticos) en los últimos 12 meses?<br><br>Qué tipo de práctica personal utilizó? _______________	□ 1. Si (Preguntar D10b)<br>□ 2. No (FINAL)<br>□ 8. No sabe (FINAL)<br>□ 9. No contesta (FINAL)	□ 00. Ninguna<br>__ __ veces<br>□ 77. No corresponde<br>□ 88. No sabe<br>□ 99. No contesta	□ 1. Un problema de salud agudo, es decir, uno que duró menos de un mes?<br>□ 2. Un problema de salud crónico, es decir, uno que duró más de un mes, ya sea para tratar el problema en sí o sus síntomas?<br>□ 3. Para mejorar su bienestar<br>□ 4. Otra, por favor especificar: ________________________________<br>□ 7. No corresponde<br>□ 8. No sabe<br>□ 9. No contesta	□ 1. Muy beneficioso<br>□ 2. Algo beneficioso<br>□ 3. Para nada beneficioso<br>□ 7. No corresponde<br>□ 8. No sabe<br>□ 9. No contesta

NAFKAM International CAM Questionnaire (I-CAM-Q):
RECOMMENDED FOR USE IN STUDIES OF
COMPLEMENTARY AND ALTERNATIVE MEDICINE (CAM) --
Self-Administered Version

1. ***Visiting health care providers:*** Health problems may be attended to by a variety of complementary and conventional health care providers.

Have you seen any of the following providers in the last 12 months?	Yes	No	Number of times you saw this provider in the last 3 months?	Please indicate the *main* reason you *last* saw the provider (Check only *one*).				How helpful was it for you to see this provider? (Check only one)			
				For an acute illness/condition, one that lasted less than one month	To treat a long-term health condition (one that lasted more than one month) or its symptoms	To improve well-being	Other (Please specify the other reason)	Very	Somewhat	Not at all	Don't know
**Physician**	☐	☐	___	☐	☐	☐	___	☐	☐	☐	☐
**Chiropractor**	☐	☐	___	☐	☐	☐	___	☐	☐	☐	☐
**Homeopath**	☐	☐	___	☐	☐	☐	___	☐	☐	☐	☐
**Acupuncturist**	☐	☐	___	☐	☐	☐	___	☐	☐	☐	☐
**Herbalist**	☐	☐	___	☐	☐	☐	___	☐	☐	☐	☐
**Spiritual healer**	☐	☐	___	☐	☐	☐	___	☐	☐	☐	☐
**Specified option:** ___	☐	☐	___	☐	☐	☐	___	☐	☐	☐	☐
**Other (please specify):** ___	☐	☐	___	☐	☐	☐	___	☐	☐	☐	☐
**Other (please specify):** ___	☐	☐	___	☐	☐	☐	___	☐	☐	☐	☐

2. Complementary treatments received from physicians (MDs)

If you have not seen a physician in the past 12 months, please go to question 3.
Some physicians provide complementary, as well as conventional treatments

Have you received any of the following complementary treatments from a physician in the last 12 months?	Yes	No	Number of times you received this treatment in the last 3 months?	Please indicate the *main* reason you *last* received this treatment (Check only *one*).				How helpful was it to receive treatment from the physician? (Check only one)			
				For an acute illness/condition, one that lasted less than one month	To treat a long-term health condition (one that lasted more than one month) or its symptoms	To improve well-being	Other (Please specify the other reason)	Very	Somewhat	Not at all	Don't know
**Manipulation**	☐	☐	____	☐	☐	☐	____________	☐	☐	☐	☐
**Homeopathy**	☐	☐	____	☐	☐	☐	____________	☐	☐	☐	☐
**Acupuncture**	☐	☐	____	☐	☐	☐	____________	☐	☐	☐	☐
**Herbs**	☐	☐	____	☐	☐	☐	____________	☐	☐	☐	☐
**Spiritual healing**	☐	☐	____	☐	☐	☐	____________	☐	☐	☐	☐
**Specified option:** ____________	☐	☐	____	☐	☐	☐	____________	☐	☐	☐	☐
**Other (please specify):** ____________	☐	☐	____	☐	☐	☐	____________	☐	☐	☐	☐

3. Use of Herbal Medicine and Dietary Supplements, including tablets, capsules and liquids.

For each category below, please list up to three products you have used in the last 12 months.	Do you currently use this product? Yes	No	Please indicate the *main* reason that applies to your *last* use (Check only *one*). For an acute illness/condition, one that lasted less than one month	To treat a long-term health condition (one that lasted more than one month) or its symptoms	To improve well-being	Other (Please specify)	How helpful did you find this product? (Check only one) Very	Somewhat	Not at all	Don't know
**Herbs/Herbal Medicine**										
______	☐	☐	☐	☐	☐	______	☐	☐	☐	☐
______	☐	☐	☐	☐	☐	______	☐	☐	☐	☐
______	☐	☐	☐	☐	☐	______	☐	☐	☐	☐
**Vitamins/Minerals**										
______	☐	☐	☐	☐	☐	______	☐	☐	☐	☐
______	☐	☐	☐	☐	☐	______	☐	☐	☐	☐
______	☐	☐	☐	☐	☐	______	☐	☐	☐	☐
**Homeopathic remedies**										
______	☐	☐	☐	☐	☐	______	☐	☐	☐	☐
______	☐	☐	☐	☐	☐	______	☐	☐	☐	☐
______	☐	☐	☐	☐	☐	______	☐	☐	☐	☐
**Other Supplements**										
______	☐	☐	☐	☐	☐	______	☐	☐	☐	☐
______	☐	☐	☐	☐	☐	______	☐	☐	☐	☐
______	☐	☐	☐	☐	☐	______	☐	☐	☐	☐

4. Self Help Practices

Have you used any of the following self-help practices in the last 12 months?	Yes	No	Number of times you used this practice in the last 3 months?	Please indicate the *main* reason that applies to your *last* use of the self-help practice (Check only *one*). For an acute illness/condition, one that lasted less than one month	To treat a long-term health condition (one that lasted more than one month) or its symptoms	To improve well-being	Other (Please specify the other reason)	How helpful did you find this self-help practice? (Check only one) Very	Somewhat	Not at all	Don't know
**Meditation**	☐	☐	___	☐	☐	☐	___	☐	☐	☐	☐
**Yoga**	☐	☐	___	☐	☐	☐	___	☐	☐	☐	☐
**Qigong**	☐	☐	___	☐	☐	☐	___	☐	☐	☐	☐
**Tai Chi**	☐	☐	___	☐	☐	☐	___	☐	☐	☐	☐
**Relaxation techniques**	☐	☐	___	☐	☐	☐	___	☐	☐	☐	☐
**Visualization**	☐	☐	___	☐	☐	☐	___	☐	☐	☐	☐
**Attended traditional healing ceremony**	☐	☐	___	☐	☐	☐	___	☐	☐	☐	☐
**Praying for own health**	☐	☐	___	☐	☐	☐	___	☐	☐	☐	☐
**Specified option:** ___	☐	☐	___	☐	☐	☐	___	☐	☐	☐	☐
**Other (please specify):** ___	☐	☐	___	☐	☐	☐	___	☐	☐	☐	☐
