# Supplementary material for: Association of significant risk perception with the use of complementary and alternative medicine: A cross-sectional study in Hispanic patients with rheumatoid arthritis
Source: PLoS One. 2020 Aug 13;15(8):e0237504. doi: 10.1371/journal.pone.0237504 (PMC7425852; doi:10.1371/journal.pone.0237504)
Supplement: Attachment — Submitted filename: Assosciations of risk perception RA_CrossSection_PLOSOne_7May2020.docx [file pone.0237504.s003.docx]

This manuscript presents a cross-sectional study investigating the association of risk perception (RP) among rheumatoid arthritis (RA) patients using complementary and alternative medicine (CAM) in a Hispanic RA outpatient population. Through interviewing a consecutive sample of patients at this single centre, they attempted to evaluate risk perception along with other factors which they believed to be associated with influencing CAM use. The authors identified limited RP-associated literature pertaining to rheumatic disease and therefore a gap in the evidence to address. The conclusion of this study appears to indicate that RP was associated to recent (within 3 month) CAM use in Hispanic RA outpatients. The strengths of this study were a clear aim, following a well-documented & reproducible methodology and appropriate sub-analyses given the included population.

As above, the authors have presented a reproducible study that investigated an area of interest to the profession. Overall this was well put together and this reviewer would recommend the editor to accept this submission pending revision pertaining to caveats outlined below.

Major revisions

- Abstract results refer to sample with prayer included as CAM. Prayer is not usually referred to as CAM, though the authors do make assertions that their population may be influenced by it. Suggest focus of the paper should be related to the prayer excluded sample. This is further supported by the I-CAM-Q not having any reference to prayer and also only being requested from patients who were in the prayer excluded CAM group in this study.
- While the authors conclusion does seem to reflect the results they found (minus that access to a federal health care system was found to be associated on both analyses as on Line 359; Discussion), as per their sample size calculation section (Line 214-215), this sample is powered to 0.74 in a one-tailed test. Therefore the conclusion should be amended to provide more uncertainty regarding these associations and suggest further investigation is necessary with a larger comparative sample (non-CAM user).
- Given the sample size calculation section (Line 209-215), it would be helpful to provide an explanation as to why the full sample was not collected (assuming an equal distribution of the calculated 220 patient, i.e. 110 in each group).
- Table 1 (Line 143) may not be set out in line with the usual journal criteria. A mix between sentence case and bullet points makes it difficult to follow the contents of this table. The authors have also chosen to use “*” as their bullet point which is usually associated with footnotes to tables. Suggest change each data point to a bullet point with an actual bullet instead of “*”.
- The Figure provided was extremely blurry and hard to interpret. A cleared image would be required to properly evaluate the information. Still, the authors have chosen to collapse if patients believed CAM was “very helpful” or “somewhat helpful” in the graph. Given these are very different responses, it may be helpful to have these represented as separate categories. It seems fine to collapse them in text (Line 353-357).
- As per above, it might be the quality of the Figure, but it seems in the "complementary therapies received from physicians” graph, the joint manipulation bar is thinner that everything else. This reviewer is unclear if there is missing/cut off information or if this is just a sizing error.

Minor revisions

- Acronyms should not start sentences (e.g. Line 34).
- On Line 76-77, the authors provide the WHO definition of CAM as “a broad set of health care practices that are not part of the country´s own tradition and are not integrated into the dominant healthcare system”. The definition appears to refer to CAM being defined based on country-specific views of mainstream medicine. However on Line 81-84, the authors say 3 certain CAM approaches are ingrained in certain countries and then say that these methods “may not be appropriately classified under the WHO definition”. This reviewer is unclear on the message the authors are trying to convey.
- The explanation of the Pain-Visual Analogue Scale (VAS) and overall disease-VAS (Line 155-162) was extremely confusing in terms of which end (left vs right) was equal to 0 or 100. It is understandable that the authors used a referenced method, but it would be helpful if the reader could be assured that the scoring clinician was not confused by this. Possible re-iteration that the clinician scoring this was trained it how to do this on Line 134-135.
- As from the point above, it is unclear why the Adherence-VAS (Line 163-165) was not explained to the same detail as the VAS in the section above (Line 155-162).
- On Line 173 the authors name the section RP Questionnaire (RPQ). As this is the name of a tool used, spelling it out instead of putting an acronym on an acronym may be better.
- On Line 247-248, it would be helpful to have some indication on the type of patient who declined an interview seeing as they consisted 7.5% of the total sample.
- In Table 2 (Line 265) and Table 3 (Line 295), RP is defined as risk score. For sake of consistency, suggest changing to “risk perception score”.
- On Line 288 the authors refer to a PR score, which is assumed to actually be RP given the rest of the manuscript.
- On Lines 300-302, the authors indicate Table 4 shows “significant RP was associated with CAM use, female sex, years of formal education …”. This seems to imply the testing of RP as the dependent variable instead of CAM. This does not seem to align with the title of Table 4 which appears to imply CAM was the dependent variable.
- On Line 423 the authors use the word “finally” and then a sentence later (Line 430) they use “finally” again. Suggest a different word on Line 423.
- On Line 448 the authors refer to the agent “thunder god wine”. This may be interpreted as a colloquial term for this treatment. This reviewer suggests maybe referring to it by its scientific name “Tripterygium wilfordii (thunder god wine)”.
- On Line 482-484 the authors refer to depression and health literacy being associated with CAM use. While they did use education years as a potential surrogate, maybe an explanation as to why this was not investigated may be prudent.
